# Development of a lesbian, gay, bisexual, and transgender cultural competence scale for nurses in South Korea: a methodological study

**DOI:** 10.4069/whn.2024.06.19

**Published:** 2024-06-28

**Authors:** Min Kyung Kim, Hye Young Kim

**Affiliations:** 1 Department of Nursing, Catholic Sangji College, Andong, Korea; 2 College of Nursing, Keimyung University, Daegu, Korea

**Keywords:** Culture, Nurses, Sexual and gender minorities

## Abstract

**Purpose:**

This study was conducted to develop a cultural competence scale for nurses regarding the lesbian, gay, bisexual, and transgender (LGBT) community and to test its validity and reliability.

**Methods:**

The study adhered to the 8-step process outlined by DeVellis, with an initial set of 25 items derived through a literature review and individual interviews. Following an expert validity assessment, 24 items were validated. Subsequently, a preliminary survey was conducted among 23 nurses with experience caring for LGBT patients. Data were then collected from a final sample of 322 nurses using the 24 items. Item analysis, item-total score correlation, examination of construct and convergent validity, and reliability testing were performed.

**Results:**

The item-level content validity index exceeded .80, and the explanatory power of the construct validity was 63.63%. The factor loadings varied between 0.57 and 0.80. The scale comprised five factors: cultural skills, with seven items; cultural awareness, with five items; cultural encounters, with three items; cultural pursuit, with three items; and cultural knowledge, with three items; totaling 21 items. Convergent validity demonstrated a high correlation, affirming the scale’s validity. Internal consistency analysis yielded an overall reliability coefficient of 0.97, signifying very high reliability. Each item is scored from 1 to 6 (total score range, 21–126), with higher scores reflecting greater cultural competence in LGBT care.

**Conclusion:**

This scale facilitates the measurement of LGBT cultural competence among nurses. Therefore, its use should provide foundational data to support LGBT-focused nursing education programs.

## Introduction

Diverse cultures coexist and are accepted in South Korea (hereafter referred to as Korea); however, lesbian, gay, bisexual, and transgender (LGBT) individuals often exist on the margins as a minority within a predominantly heterosexual society [1]. The social stigma surrounding LGBT identities leads some individuals to conceal their orientation or gender identity, resulting in limited and potentially inaccurate statistical data on the LGBT population [2]. Estimates of the number of LGBT people in Korea vary, with figures ranging from 1.81 million to 2.69 million [3].

As a social minority, LGBT people are sometimes associated with certain diseases and can be targets of hatred due to negative perceptions and prejudice [4]. Among other social minority groups in Korea, such as North Korean refugees, people with disabilities, and foreign workers, LGBT people encounter the most intense negative stereotypes and prejudice [5]. According to the 2017–2022 World Value Survey 7th Wave, 79.6% of Koreans indicated they “would not want to have a homosexual as a neighbor,” a sentiment more prevalent than in China at 70.8%, Japan at 26.4%, the United States at 12.7%, and Germany at 6.4% [6]. Despite an increase in public opinion about LGBT individuals and a broadening understanding of different sexual orientations, Korea’s acceptance of homosexuality remains the fourth lowest among the 36 countries of the Organisation for Economic Co-operation and Development [7]. Furthermore, although the visibility of the LGBT population is growing, health research on LGBT individuals in Korea remains scarce [8]. In contrast, the U.S. National Institute of Health recognized LGBT people as a health disparity population in 2016, and the U.S. Department of Health and Human Services included LGBT health as a focus area in their Healthy People 2020 initiative to improve health in the United States [9]. This reflects an increasingly pressing need for healthcare providers to offer culturally competent services to address health inequities affecting LGBT individuals.

Culture refers to the integrated aspects of human behavior, including language, thought, behavior, customs, and beliefs. These elements influence individuals’ health beliefs, perceptions of illness, healthcare utilization behaviors and attitudes, and understanding and acceptance of care [10]. Consequently, cultural competence—the ability of healthcare providers to effectively care for and accept individuals from diverse cultural backgrounds—is of paramount importance in healthcare settings. Cultural competence enables nurses to understand and respect the values, attitudes, and beliefs of patients from various cultures [11]. Therefore, it is imperative for nurses to possess cultural competence to engage with clients from diverse backgrounds without prejudice and discrimination and to address their diverse needs [12].

Limited research on LGBT health is available within the nursing literature, with only a few publications addressing LGBT health issues [13]. A lack of understanding among nurses regarding LGBT individuals may impede their ability to deliver culturally competent care. Various instruments have been employed in previous studies to measure nurses’ cultural competence concerning LGBT populations. These include the Attitudes Toward Lesbians and Gay Men Scale by Herek [14], the Knowledge About Homosexuality Questionnaire by Harris et al. [15], and the Gay Affirmative Practice Scale by Crisp [16]. However, these studies generally have not represented all LGBT people, and only a limited number of cultural competence dimensions—such as knowledge, attitudes, behaviors, and beliefs—have been explored in the existing research.

Therefore, the cultural competence instruments developed thus far face challenges in capturing the social understanding and cultural characteristics of LGBT individuals. Consequently, the measurement of cultural competence regarding LGBT populations has been limited, especially in nursing research. This study was conducted to develop an instrument designed to measure LGBT cultural competence among nurses. By grounding the measure in the concept and components of cultural competence, the goal is to promote comprehensive healthcare delivery, improve the quality of healthcare services, and reduce discrimination and prejudice.

## Methods

**Ethics statement:** This study was approved by the Institutional Review Board of Keimyung University (40525-202105-HR-016-02). Informed consent was obtained from the participants.

### Study design

This methodological study was designed to develop a measure of LGBT cultural competence among nurses and to evaluate its validity and reliability.

### Development of the instrument

#### Conceptual framework

This study utilized the conceptual framework of the Campinha-Bacote model [17], which delineates the process of cultural competence in healthcare service delivery. This model integrates five components: cultural awareness, cultural knowledge, cultural skill, cultural encounters, and cultural desire, which are essential for nurses providing care to LGBT individuals. The Campinha-Bacote model [17] describes the practice in which healthcare providers deliver inclusive care, recognizing that cultural diversity extends beyond race and nationality to include sexual orientation and gender identity [18].

### Study procedures

**Figure 1.**
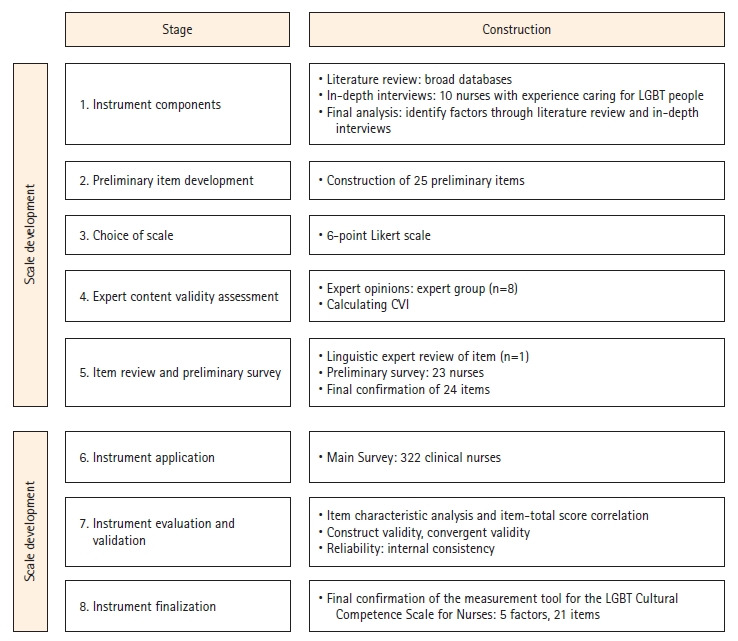
Development process of the LGBT Cultural Competence Scale for Nurses. CVI, Content validity index; LGBT: lesbian, gay, bisexual, and transgender.

This study followed the eight-step tool development process outlined by DeVellis [19]. The specific research process is as follows (Figure 1).

#### Step 1: instrument components

A literature review and individual in-depth interviews were conducted to identify components of the instrument. The literature review utilized several databases, including KISS (Korean Studies Information Service System), RISS (Research Information Sharing Service), KCI (Korea Citation Index), ClinicalKey for Nursing, PubMed, EMBASE, and ProQuest, to search for relevant published articles and completed theses. The primary search terms included “LGBT,” “homosexuality,” “lesbian,” “gay,” “transgender,” “cultural competence,” “nurse,” “nursing,” and “Bisexual.” Following the initial findings from the literature review, specific criteria were established for conducting individual in-depth interviews. These criteria required nurses to have a minimum of 1 year of clinical experience at a university hospital and to have cared for at least one patient who disclosed their sexuality for treatment purposes, taking into account the special considerations for LGBT patients. Participants for the in-depth interviews were recruited through a social network system (SNS), with the purpose of the research clearly communicated. The interviews were conducted face-to-face, lasting between 1 and 1.5 hours each. The interview process continued until data saturation was achieved, indicated by the repetition of content and the absence of new information. In total, 10 nurses participated in these interviews.

#### Step 2: preliminary item development

Preliminary questions were formulated based on the components of LGBT cultural competency for nurses, identified through a literature review and individual in-depth interviews.

#### Step 3: choice of scale

In this study, we employed a 6-point Likert scale to eliminate neutral responses. This approach facilitated a clearer understanding of the respondents’ attitudes and reduced the potential for distortion when interpreting the results.

#### Step 4: expert content validity assessment

To ensure that the preliminary instrument, which comprised a set of initial questions, was effectively organizing the content intended to be measured, we carried out a content validation with a panel of experts. Based on Lynn’s recommendations [20] for participant numbers in content validation studies, we engaged eight experts: three nursing professors with expertise in psychiatric nursing and multiculturalism, one medical school professor with teaching experience regarding LGBT issues, one social work professor experienced in tool development, and three practitioners holding master’s degrees and possessing at least 8 years of clinical experience in caring for LGBT patients. We determined the item-level content validity index (I-CVI) by calculating the proportion of experts providing certain responses regarding the relevance of each item. Items with an I-CVI of .80 or higher were selected [21]. Additionally, we considered preliminary items appropriate if the scale-level content validity index (S-CVI), obtained by dividing the sum of the I-CVI scores by the total number of items, was .90 or higher. Subsequently, we refined and expanded the questions to incorporate the insights gained from the expert consultations.

#### Step 5: item review and preliminary survey

We conducted a preliminary survey involving 23 nurses with experience in caring for LGBT individuals. During this survey, participants provided feedback on the initial questions and general characteristics. The survey was then refined to improve the clarity of each question, the response time, the layout of the questionnaire, and the length of the questions, as well as to add content when necessary. Following the results of the preliminary survey, an expert with a master’s degree in Korean studies and over 10 years of experience as a Korean language instructor at a university assessed the overall grammar and vocabulary of the survey items.

#### Step 6: instrument application

**Study participants:** The participants in this study were nurses who had a minimum of 1 year of clinical experience. Information about the study’s purpose and procedures, as well as a link to the survey, was disseminated through an SNS used by nurses and nursing students. Drawing on previous research that addressed the appropriate sample size for factor analysis [22], we aimed for 300 participants, estimating a 10% dropout rate. Consequently, 333 individuals were selected through convenience sampling. After discarding 11 responses considered inadequate, the final dataset comprised 322 responses.

**Data collection:** An online survey was conducted from February 23 to March 1, 2022. The purpose and procedures of the study, along with a link to the survey, were posted on online community bulletin boards and SNSs, including Instagram, Facebook, and KakaoTalk. These platforms are frequented by nurses and nursing students employed at hospitals in Korea. To encourage participation in the survey, respondents were offered a mobile voucher valued at approximately 7 US dollars.

#### Step 7: instrument evaluation

Descriptive statistics were calculated for item analysis. To assess construct validity, exploratory factor analysis was conducted. Convergent validity was evaluated using the shortened version of the Nurses’ Cultural Competence Instrument, developed by Chae and Park [23]. The Cronbach alpha was calculated to determine the internal consistency of the items within the tool, ensuring a consistent measurement of the intended content.

#### Step 8: instrument finalization

The instrument was optimized by eliminating items that compromised its validity and reliability. Following this removal, the scale was ready for use.

### Data analysis

The collected data were analyzed using SPSS ver, 24.0 (IBM Corp., Armonk, NY, USA). The methods of analysis included the following.

1) Descriptive statistics were used to analyze the general characteristics of the study participants.

2) The I-CVI and S-CVI/average proportion were employed to confirm the content validity of the preliminary tool.

3) To assess the suitability of the collected data for exploratory factor analysis, we utilized the Kaiser-Meyer-Olkin (KMO) measure of sampling adequacy and the Bartlett test of sphericity. A KMO value exceeding .80 was considered appropriate for factor analysis. Furthermore, a rejection of the null hypothesis in the Bartlett test of sphericity suggests that the data are appropriate for factor analysis. We also employed the Varimax method for factor rotation. Items with eigenvalues over 1.0 and factor loadings greater than .50 were selected to determine the number of factors.

4) The Pearson correlation coefficient was used to assess convergent validity. A coefficient of .10 to .30 was interpreted as indicating a low correlation, .30 to .50 a moderate correlation, and .50 or higher a high correlation [24].

5) The internal consistency reliability of the developed tool was assessed using the Cronbach alpha.

6) Content analysis was employed to examine the qualitative data that had been collected.

## Results

### Step 1: instrument components

The literature review and individual in-depth interviews yielded four dimensions and 12 attributes, from which the components of nurses’ LGBT cultural competence were identified.

### Step 2: preliminary items

Based on the dimensions, attributes, and indicators identified in the prior step, a total of 25 preliminary items were derived, distributed across the four dimensions: 10 items pertained to awareness and knowledge, two to experience, eight to skills, and five to motivation.

### Step 3: choice of scale

We opted to use a Likert scale, which is a commonly used method in the social sciences to measure opinions, beliefs, and attitudes. To avoid neutral responses, a 6-point Likert scale with a range of 1 (“not at all”) to 6 (“very much”) was chosen.

### Step 4: expert content validity

The I-CVI values ranged from .88 to 1.00. The S-CVI was determined by dividing the sum of the I-CVIs by the total number of items, yielding a value of .95. This met the criterion for acceptability, established at .90 or higher.

### Step 5: item review and preliminary survey

To evaluate the items prior to the main survey, a preliminary survey was administered to 23 nurses with experience caring for sexual minorities. The completion time for the survey varied from 4 to 25 minutes, with an average of 8.93 minutes. The overall comprehension of the survey items was rated at 3.83±0.94. The layout of the items in the questionnaire was rated 4.13±0.76, and the appropriateness of the length of the items received a score of 3.87±0.76 (Table 1). After a review by an expert in Korean studies, minor revisions were made to the grammar and the use of vocabulary particles in the items, resulting in a finalized set of 24 items.

### Step 6: main survey findings

#### General characteristics of the participants

Of the 322 participants in this study, 22 were male (6.8%) and 300 (93.2%) were female. The most common age group was 30 to 39 years old, representing 203 participants (63.1%). The majority were married, accounting for 172 individuals (53.4%). The highest level of education for most was a bachelor’s degree, held by 254 participants (78.9%). Regarding religion, 195 participants (60.6%) reported having none. In terms of work experience, the largest group consisted of those with more than 5 but less than 10 years, totaling 124 (38.5%). The position most frequently held was that of a staff nurse, by 272 participants (84.4%), and the most common work department was the general ward, with 237 individuals (73.6%) (Table 2).

### Step 7: instrument evaluation results

#### Correlation analysis between item characteristics and item-total scores

The findings regarding the validity of the items, as measured by the mean, standard deviation, skewness, and kurtosis of each item, are as follows. The mean values of the items varied from 3.08 to 4.52, while the standard deviation ranged from 1.15 to 1.67, which is not considered extreme. Additionally, the skewness and kurtosis for all items fell within the ±2.00 range, suggesting that each item conformed to the assumption of normality. To assess the contribution of the 24 selected items, we examined the item-total score correlation coefficient and the Cronbach alpha value when items were deleted. Items that correlated less than .30 with the total score were deemed to have a low contribution to the scale domain [25]. The correlation coefficients between the items and the total score ranged from .37 to .67, with no items falling below .30, and the Cronbach alpha value for all items was .92. Consequently, an exploratory factor analysis was conducted using the 24 items.

#### Construct validity: exploratory factor analysis

For the 24 items selected through item characterization, exploratory factor analysis was performed four times to determine the loading structure and the factors associated with each item. The KMO measure of sampling adequacy was .90, which exceeds the recommended threshold of .80 [26]. Additionally, the Bartlett test of sphericity indicated statistical significance (*χ*^2^=2,986.48 [degrees of freedom, 210], *p*<.001), confirming the appropriateness of the data for factor analysis. Five factors were extracted with eigenvalues of 1.0 or higher, which accounted for 63.63% of the variance. The factor loadings ranged from .57 to .80, and the communalities for each item varied from .51 to .76, all exceeding the predetermined cutoff value (Table 3).

Following exploratory factor analysis, a total of 21 items were identified across five factors. These factors were as follows: “cultural skills,” including seven items; “cultural awareness,” comprising five items; “cultural experience,” with three items; “cultural pursuit,” also with three items; and “cultural knowledge,” with three items.

#### Convergent validity test findings

The Pearson correlation coefficient between the 21 items of the developed scale and the 14 items of the Nurses’ Cultural Competency Measurement Tool was .70 (*p*<.001), indicating a high positive correlation. Furthermore, the Pearson correlation coefficients between the Nurses’ Cultural Competency Measurement Tool and the subfactors of the developed scale demonstrated significance and moderate strength: .39 (*p*<.001) for cultural skills, .60 (*p*<.001) for cultural awareness, .45 (*p*<.001) for cultural experience, .64 (*p*<.001) for cultural pursuit, and .62 (*p*<.001) for cultural knowledge. Thus, the convergent validity was deemed acceptable (Table 4).

#### Reliability testing: assessment of internal consistency

Considering that a reliability coefficient of .60 or higher is deemed reliable [24], the overall Cronbach alpha for the 21 items of the final instrument was excellent, at .97. The specific Cronbach alpha values were .87 for cultural skills, .81 for cultural awareness, .75 for cultural experience, .73 for cultural pursuits, and .68 for cultural knowledge.

### Step 8: instrument finalization

The instrument was finalized with 21 items, categorized into five factors: cultural skills (seven items), cultural awareness (five items), cultural experiences (three items), cultural pursuits (three items), and cultural knowledge (three items). The final instrument uses a scale ranging from 1, indicating “not at all,” to 6, signifying “very much.” The summed total score ranges from 21 to 126, with higher scores denoting greater LGBT cultural competence (Supplementary Material 1).

## Discussion

Based on the conceptual framework of the Campinha-Bacote model of the process of cultural competence in the delivery of healthcare services [17], this study developed items for an LGBT cultural competence scale for nurses. It also tested the validity and reliability of these items to establish the final scale. The resulting scale comprises 21 items across five factors: cultural skills, cultural awareness, cultural experience, cultural pursuit, and cultural knowledge. These factors reflect the shared meanings of the subfactors.

Among the five identified factors, cultural skills exhibited the highest explanatory power (36.59%). This factor comprises items evaluating the overall nursing performance of skills and practices necessary for caring for LGBT individuals, as well as the ability to provide care in a comfortable and safe environment. Cultural awareness displayed the second-highest explanatory power, at 8.59%. Along similar lines, a previous instrument developed by Crisp [16] included items measuring attitudes, prejudices, and beliefs about LGBT people. Nurses have reported that their personal beliefs about LGBT individuals sometimes lead to feelings of unfamiliarity and discomfort in providing care [27]. Furthermore, nurses must recognize the diversity of the population in their efforts to understand their own values, beliefs, attitudes, and biases and to acknowledge the diverse cultures of others [28]. As such, nurses’ cultural awareness of LGBT people is important, and this factor includes preconceived notions about the population, the cultivation of unbiased positive perceptions, and the evaluation of personal beliefs. The next factor, cultural experience, had an explanatory power of 7.76%. It is composed of questions regarding perceptions of, experiences with, and treatment of LGBT individuals. This factor underscores nurses’ roles and responsibilities in delivering quality nursing care through understanding, respect, and a professional approach to LGBT patients. In turn, cultural pursuits demonstrated an explanatory power of 5.65%. It includes questions about the willingness and motivation to actively seek education and training to better understand LGBT people. Finally, cultural knowledge demonstrated an explanatory power of 5.02%. This factor involves understanding the concept and definition of LGBT, as well as the social context of this population’s healthcare needs, illnesses, and challenges in accessing healthcare. This differs from the concept of cultural knowledge as a curriculum in which healthcare providers learn about the basic culture and values of different cultural groups [17,29]. A lack of knowledge about LGBT individuals among nurses can lead to increased uncertainty in nursing care, which may impact the quality of care [27]. However, this study has limitations due to its focus on clinical nurses, which necessitates caution when generalizing the results. Additionally, despite the anonymity of the survey, some nurses may have selected responses to conform to social expectations.

The LGBT cultural competence scale for nurses, developed in this study, may be utilized in nursing education to evaluate the cultural competence levels of nurses regarding LGBT care. It can also measure changes after educational interventions. Furthermore, this scale can contribute to establishing a systematic nursing education system, as the measurement results may be useful in planning and implementing educational programs for nurses. Additionally, the instrument can be employed to assess the LGBT cultural competency of both new and experienced nurses on an ongoing basis. This continuous assessment approach, rather than a one-time evaluation, can improve the effectiveness of training programs. In the context of nursing practice, this tool is relevant for nurses who provide care to LGBT individuals in clinical settings. By accurately measuring nurses’ LGBT cultural competence in these environments, the tool is expected to improve the quality of nursing care, ensuring safe and comfortable care for LGBT patients. Finally, this study identified the attributes of nurses’ LGBT cultural competence through a literature review and qualitative data analysis, which were integral to the development of the instrument. The findings from this process can be used to support future research on LGBT issues in nursing.

## Figures and Tables

**Table 1.** Results of item analysis (N=23)

Category	Range	Mean±SD
Time required to complete the survey (minute)	4–25	8.93±5.10
Level of clarity of the questions	1–5	3.83±0.94
Appropriateness of layout	1–5	4.13±0.76
Appropriateness of question length	1–5	3.87±0.76

**Table 2.** General characteristics of participants in the main survey (N=322)

Characteristic	Categories	N (%)
Sex	Female	300 (93.2)
	Male	22 (6.8)
Age (year)	20–29	78 (24.2)
	30–39	203 (63.1)
	40–49	38 (11.8)
	≥50	3 (0.9)
Marriage status	Single	147 (45.7)
	Married	172 (53.4)
	Others	3 (0.9)
Level of education	≤Junior college	42 (13.0)
	University	254 (78.9)
	≥Graduate school	26 (8.1)
Religion	Protestant	69 (21.4)
	Roman Catholic	29 (9.0)
	Buddhist	29 (9.0)
	None	195 (60.6)
Position	Staff nurse	272 (84.4)
	Charge nurse	35 (10.9)
	Head nurse	9 (2.8)
	Others	6 (1.9)
Work experience (year)	≤1–4	101 (31.4)
	5–9	124 (38.5)
	10–19	91 (28.2)
	≥20	6 (1.9)
Department	General ward	237 (73.6)
	Outpatient	17 (5.3)
	Emergency room	12 (3.7)
	Intensive care unit	36 (11.2)
	Others	20 (6.2)

**Table 3.** Results of exploratory factor analysis (N=322)

Item No.	Communality	F1	F2	F3	F4	F5
1	.63	.34	.65	.23	.17	.05
2	.61	.03	.77	.13	−.01	.06
3	.64	.19	.76	.04	.13	.10
4	.66	.01	.70	−.01	.42	.06
5	.58	.35	.64	.17	.02	.13
6	.60	.28	.20	.09	.17	.66
7	.65	.41	−.01	.10	.05	.69
8	.68	−.05	.09	.28	.28	.72
9	.68	.18	.15	.66	.32	.29
10	.65	.13	.11	.78	.09	.08
11	.68	.13	.12	.80	−.04	.10
12	.51	.57	.21	.03	−.09	.36
13	.63	.69	.21	.06	.32	.07
14	.60	.66	.22	.18	.16	.23
15	.65	.67	.15	.34	.25	−.06
16	.60	.62	.05	.09	.44	.12
17	.64	.67	.08	−.01	.37	.22
18	.69	.71	.19	.36	−.01	.17
19	.57	.26	.21	.35	.58	.08
20	.76	.23	.20	.17	.77	.20
21	.65	.42	.12	−.14	.61	.27
22	.63	.34	.65	.23	.17	.05
23	.61	.03	.77	.13	−.01	.06
24	.64	.19	.76	.04	.13	.10
Eigenvalues		7.68	1.80	1.63	1.19	1.05
Variance (%)		36.69	8.6	7.8	5.7	5.0
Cumulative variance (%)		36.6	45.2	52.9	58.6	63.6
Kaiser-Meyer-Olkin=.90						
Bartlett test of sphericity: χ2=2,986.48 (degrees of freedom, 210), *p*<.001						

**Table 4.** Results regarding convergent validity (N=322)

Nurses’ cultural competency	Nurses’ cultural competency towards sexual minorities, r (*p*)					
	Total	Culture skill	Culture awareness	Culture experience	Culture pursuit	Culture knowledge
Total	.70 (<.001)	.39 (<.001)	.60 (<.001)	.45 (<.001)	.64 (<.001)	.62 (<.001)
Sensitivity	.64 (<.001)	.38 (<.001)	.49 (<.001)	.36 (<.001)	.63 (<.001)	.54 (<.001)
Knowledge	.51 (<.001)	.26 (<.001)	.45 (<.001)	.50 (<.001)	.36 (<.001)	.47 (<.001)
Awareness	.61 (<.001)	.31 (<.001)	.59 (<.001)	.30 (<.001)	.60 (<.001)	.54 (<.001)
Skill	.59 (<.001)	.35 (<.001)	.50 (<.001)	.38 (<.001)	.52 (<.001)	.53 (<.001)

## Supplementary materials

Further details on supplementary materials are presented online (available at https://doi.org/10.4069/whn.2024.06.19).
