# Supplementary material for: Development of a lesbian, gay, bisexual, and transgender cultural competence scale for nurses in South Korea: a methodological study
Source: Womens Health Nurs. 2024 Jun 28;30(2):107–16. doi: 10.4069/whn.2024.06.19 (PMC11237366; doi:10.4069/whn.2024.06.19)
Supplement: Supplementary Material 1. [file whn-2024-06-19-Supplementary-Material-1.pdf]

Supplementary Material 1. 최종 확정된 간호사의 성소수자 문화역량 측정도구

① 전혀 그렇지 않다 ②그렇지 않다 ③ 약간 그렇지 않다 ④ 약간 그렇다 ⑤ 그렇다 ⑥ 매우 그렇다

차원	번호	문항	①	②	③	④	⑤	⑥
문화적 기술	1	나는 대중매체나 교육 등을 통해 성소수자를 접해본 적이 있다.	①	②	③	④	⑤	⑥
	2	나는 성소수자가 성정체성이나 성적 지향을 밝혀도, 편안하게 간호 기술을 수행할 수 있다.	①	②	③	④	⑤	⑥
	3	나는 성소수자를 간호할 때, 혈액매개 감염으로부터 안전한 간호를 수행할 수 있다.	①	②	③	④	⑤	⑥
	4	나는 성소수자와 적절한 치료적 의사소통을 할 수 있다.	①	②	③	④	⑤	⑥
	5	나는 성소수자에게 간호사정(간호정보조사 및 신체사정 등)을 자연스럽게 수행할 수 있다.	①	②	③	④	⑤	⑥
	6	나는 성소수자에게 일반 대상자와 같이 동일하게 간호를 제공할 수 있다.	①	②	③	④	⑤	⑥
	7	나는 성소수자를 간호하기에 충분한 능력이 있다.	①	②	③	④	⑤	⑥
문화적 인식	8	나는 성소수자가 정상적이라고 생각한다.	①	②	③	④	⑤	⑥
	9	나는 성소수자가 나와 같다고 느낀다.	①	②	③	④	⑤	⑥
	10	나는 성소수자들이 성적으로 문란하다고 생각하지 않는다.	①	②	③	④	⑤	⑥
	11	나는 성소수자들이 성정체성이나 성적지향을 드러내는 것이 자연스럽다고 느껴진다.	①	②	③	④	⑤	⑥
	12	성소수자를 간호하는 것은 나의 신념(종교, 도덕 등)과 맞다.	①	②	③	④	⑤	⑥
문화적경험	13	나는 성소수자를 간호하기 위해 필요한 지식을 알고 있다.	①	②	③	④	⑤	⑥
	14	나는 가족, 친구, 지인, 동료 등으로 성소수자와 만남을 경험한 적이 있다.	①	②	③	④	⑤	⑥
	15	나는 간호사로 근무하면서 성소수자를 환자로 만난 적이 있다.	①	②	③	④	⑤	⑥
문화적추구	16	나는 성소수자를 이해하기 위해 적극적으로 노력한다(교육, 대중매체, 도서 등).	①	②	③	④	⑤	⑥
	17	나는 성소수자 간호를 위해 교육과 훈련을 통해 필요한 지식과 기술을 배우고 싶다.	①	②	③	④	⑤	⑥
	18	나는 성소수자의 개인 정보 보호를 위해 노력한다.	①	②	③	④	⑤	⑥
문화적 지식	19	나는 성소수자에 대한 의미, 개념, 정의 등에 대해 알고 있다.	①	②	③	④	⑤	⑥
	20	나는 성소수자들이 걸릴 수 있는 다양한 질병에 대해 알고 있다.	①	②	③	④	⑤	⑥
	21	나는 성소수자들이 의료 서비스 이용에 어려움을 겪고 있다는 것을 알고 있다.	①	②	③	④	⑤	⑥
